# Induction chemotherapy for locoregionally advanced nasopharyngeal carcinoma

**DOI:** 10.1186/s40880-016-0157-4

**Published:** 2016-11-15

**Authors:** Wen-Fei Li, Lei Chen, Ying Sun, Jun Ma

**Affiliations:** Department of Radiation Oncology, State Key Laboratory of Oncology in South China; Collaborative Innovation Center for Cancer Medicine, Sun Yat-sen University Cancer Center, 651 Dongfeng Road East, Guangdong, 510060 P. R. China

**Keywords:** Nasopharyngeal carcinoma, Induction chemotherapy, Concurrent chemoradiotherapy

## Abstract

The value of adding induction chemotherapy (IC) to concurrent chemoradiotherapy (CCRT) for the treatment of locoregionally advanced nasopharyngeal carcinoma (NPC) remains unclear. In our recent article entitled “Induction chemotherapy plus concurrent chemoradiotherapy versus concurrent chemoradiotherapy alone in locoregionally advanced nasopharyngeal carcinoma: a phase 3, multicentre, randomised controlled trial” published in the *Lancet Oncology*, we reported the results of a phase III, multicenter, randomized controlled trial comparing cisplatin, 5-fluorouracil, and docetaxel (TPF) IC plus CCRT versus CCRT alone in patients with T3-4N1/TxN2-3M0 NPC (ClinicalTrials.gov registration number NCT01245959). The IC-plus-CCRT group showed significantly higher 3-year failure-free survival, overall survival, and distant failure-free survival rates than the CCRT-alone group, with an acceptable toxicity profile. Our study suggests that adding TPF IC to CCRT could increase survival rates and reduce distant failure in patients with locoregionally advanced NPC. However, long-term follow-up is required to assess the eventual efficacy and toxicity of this strategy, and a more accurate method to determine prognosis is needed to enable better tailoring of treatment strategy for individual patients.

## Background

Nasopharyngeal carcinoma (NPC) is a unique head and neck cancer with an extremely unbalanced endemic distribution [1, 2]. Radiotherapy is the primary treatment modality for non-disseminated NPC. With advances in imaging techniques, the advent of intensity-modulated radiotherapy (IMRT), and the use of concurrent chemotherapy in patients with advanced disease, the survival rates of patients with NPC have been substantially improved [3]. The 5-year overall survival (OS) rates are high for patients with stage I–II disease (90%–100%) but unsatisfactory for patients with stage III–IVB disease (60%–85%), with distant metastasis being the major pattern of failure [4, 5].

To further improve systemic control and survival of patients with locoregionally advanced NPC, adding induction chemotherapy (IC) to concurrent chemoradiotherapy (CCRT) seems to be a promising treatment strategy. NPC is highly responsive to IC, and the advantages of IC include better tolerability, early eradication of micrometastases, and simultaneous normal tissue protection and increased tumor radiosensitivity due to tumor shrinkage. However, the value of adding IC to CCRT remains controversial in the treatment of locoregionally advanced NPC [6–8]. In a randomized phase II study by Hui et al. [6], IC with docetaxel and cisplatin followed by CCRT resulted in a 3-year OS benefit compared with CCRT alone in patients with stage III–IVB NPC; however, in another phase II trial, by Fountzilas et al. [7], IC using cisplatin, epirubicin, and paclitaxel followed by CCRT did not significantly increase OS or progression-free survival rate compared with CCRT alone. Tan et al. [8] performed a randomized phase II/III trial that compared IC (gemcitabine, carboplatin, and paclitaxel) plus CCRT with CCRT alone in patients with stage III-IVB NPC and observed no significant survival differences between the two groups. Different trial designs and study populations make direct comparisons of these studies difficult. In three Bayesian network meta-analyses, IC plus CCRT did not significantly increase OS compared with CCRT alone [9–11] but showed a significant benefit for distant control [10, 11]. The efficacy of IC followed by CCRT in locoregionally advanced NPC still lacks solid evidence from large-scale randomized controlled trials.

## Main text

In our recent article entitled “Induction chemotherapy plus concurrent chemoradiotherapy versus concurrent chemoradiotherapy alone in locoregionally advanced nasopharyngeal carcinoma: a phase 3, multicentre, randomised controlled trial” published in the *Lancet Oncology*, we reported the results of a phase III, multicenter, randomized controlled trial at 10 institutions in China, which compared cisplatin, 5-fluorouracil, and docetaxel (TPF) IC plus CCRT vs CCRT alone in the treatment of locoregionally advanced NPC (ClinicalTrials.gov registration number NCT01245959) [12]. In this study, 480 NPC patients with stage III–IVB (except T3–4N0; according to the 7th version of staging system of Union for International Cancer Control and American Joint Committee on Cancer) disease were randomly assigned to receive IC plus CCRT (*n* = 241) or CCRT alone (*n* = 239). Both groups received 100 mg/m^2^ cisplatin every 3 weeks for three cycles concurrently with IMRT. The IC-plus-CCRT group received docetaxel (60 mg/m^2^ on d1), cisplatin (60 mg/m^2^ on d1), and 5-fluorouracil (600 mg/m^2^ per day on d1–5, 120-h infusion) every 3 weeks for three cycles before CCRT. The primary endpoint was failure-free survival rate. After a median follow-up of 45 months, the IC-plus-CCRT group showed a significantly higher 3-year failure-free survival rate (80 vs. 72%; hazard ratio [HR] = 0.68, 95% confidence interval [CI]: 0.48–0.97; *P* = 0.034), OS rate (92 vs. 86%; HR = 0.59, 95% CI: 0.36–0.95; *P* = 0.029), and distant failure-free survival rate (90 vs. 83%; HR = 0.59, 95% CI: 0.37–0.96; *P* = 0.031) than the CCRT-alone group. No significant difference was observed in 3-year locoregional failure-free survival rates between the two groups (92 vs. 89%, *P* = 0.117). During the entire treatment course, the IC-plus-CCRT group had significantly higher occurrence rate of grade 3 or 4 adverse events than the CCRT-alone group (73 vs. 54%, *P* < 0.001), but the adverse events were generally uncomplicated and manageable. We theorized that the effective TPF induction regimen, adequately powered sample size, and inclusion of patients with high-risk T3-4N1/TxN2-3M0 disease may have contributed to the positive results.

Our study showed that adding TPF IC to CCRT could significantly increase failure-free survival, OS, and distant failure-free survival rates in patients with locoregionally advanced NPC. Several randomized trials (such as NCT00201396, NCT00705627, and NCT01872962) are also evaluating the therapeutic benefits of adding different induction regimens to CCRT; we await confirmation of the value of this strategy. Moreover, our study showed that patients with T3-4N1/TxN2-3M0 NPC could benefit from TPF IC; however, exploratory analysis for covariate interaction (e.g., N1 vs. N2-3) of the treatment effect did not find any significant interaction, indicating that the effect of IC did not vary among the subgroups of patients with NPC [12]. Therefore, additional meta-analyses combining more relevant trials are needed to determine whether certain subgroups of patients with NPC could benefit more from additional IC. In the study by Chua et al. [13], patients with detectable pre-treatment Epstein-Barr virus (EBV) DNA levels appeared to benefit from IC, which indicates that the pre-treatment EBV DNA level potentially has a role as a predictive marker for IC in stratifying patients with locoregionally advanced NPC.

Our study showed that IC plus CCRT was better than CCRT alone in the treatment of patients with locoregionally advanced NPC; however, whether the induction-concurrent strategy is better than the concurrent-adjuvant sequence remains unclear. In the phase III trial NPC-0501, Lee et al. [14] evaluated the potential therapeutic benefit of changing from a concurrent-adjuvant chemotherapy sequence to an induction-concurrent chemotherapy sequence. Unadjusted comparisons of induction sequences versus adjuvant sequences did not reach statistical significance, but adjusted comparisons indicated favorable improvements by induction sequence [14]. In addition, whether an induction-concurrent sequence is sufficient for patients at high risk of distant failure still needs further evaluation. In our study, 14% of patients with N2-3 disease in the TPF IC-plus-CCRT group still experienced distant metastasis. Wang et al. [15] showed that NPC patients with persistently detectable plasma EBV DNA after curative radiotherapy have a higher rate of treatment failure and short survival, and administration of adjuvant chemotherapy in these patients could significantly reduce distant failure, suggesting that individualized treatment based on EBV DNA is important to further improve the survival of patients at high risk of distant failure.

Some issues still need to be addressed regarding the use of IC in the treatment of NPC. First, it remains uncertain whether TPF is the best regimen to be recommended. Recently, Zhang et al. [16] showed that, in patients with recurrent or metastatic NPC, gemcitabine plus cisplatin (GP) significantly extended progression-free survival and OS compared with 5-fluorouracil plus cisplatin. GP is also an effective and promising regimen for NPC; thus, we conducted another phase III, randomized trial comparing GP IC plus CCRT versus CCRT alone in patients with locoregionally advanced NPC (NCT01872962), and the currently observed benefit of TPF IC might also be achieved by using GP IC. Second, the prognostic value of tumor response to IC needs further evaluation. In our study, 9% of patients had stable disease after three cycles of TPF IC. Liu et al. [17] found that an unsatisfactory tumor response (stable disease or disease progression) or detectable EBV DNA levels after IC were predictors of poor prognosis for patients with locoregionally advanced NPC. In a prospective study of children and young adults with NPC, the radiation dose during CCRT was decreased to 54 Gy in patients who achieved complete remission and 59.4 Gy in those who achieved partial remission after three cycles of IC; after a median follow-up of 30 months, none of these patients experienced locoregional recurrence [18]. These findings warrant further risk stratification and early treatment modification after IC in patients with locoregionally advanced NPC.

## Conclusions

The results of our recent phase III trial showed that, for the treatment of locoregionally advanced NPC, the addition of TPF IC to CCRT significantly increased failure-free survival, OS, and distant failure-free survival rates. We recommend TPF IC followed by CCRT for the treatment of patients with locoregionally advanced NPC. However, long-term follow-up is required to assess the eventual efficacy and toxicity of this strategy, and a more accurate method to determine prognosis is needed to enable better tailoring of treatment strategy for individual patients.
